# Treatment of distal humeral fractures using conventional implants. Biomechanical evaluation of a new implant configuration

**DOI:** 10.1186/1471-2474-11-172

**Published:** 2010-08-04

**Authors:** Markus Windolf, Edgardo Ramos Maza, Boyko Gueorguiev, Volker Braunstein, Karsten Schwieger

**Affiliations:** 1 AO Research Institute, AO Foundation, Clavadelerstrasse 8, 7270 Davos, Switzerland; 2 Hospital General Regional # 2 del Instituto Mexicano del Seguro Social, Mexico City, Mexico; 3 Department of Traumatology and Orthopaedic Surgery, University of Munich, Munich, Germany

## Abstract

**Background:**

In the face of costly fixation hardware with varying performance for treatment of distal humeral fractures, a novel technique (U-Frame) is proposed using conventional implants in a 180° plate arrangement. In this in-vitro study the biomechanical stability of this method was compared with the established technique which utilizes angular stable locking compression plates (LCP) in a 90° configuration.

**Methods:**

An unstable distal 3-part fracture (AO 13-C2.3) was created in eight pairs of human cadaveric humeri. All bone pairs were operated with either the "Frame" technique, where two parallel plates are distally interconnected, or with the LCP technique. The specimens were cyclically loaded in simulated flexion and extension of the arm until failure of the construct occurred. Motion of all fragments was tracked by means of optical motion capturing. Construct stiffness and cycles to failure were identified for all specimens.

**Results:**

Compared to the LCP constructs, the "Frame" technique revealed significant higher construct stiffness in extension of the arm (P = 0.01). The stiffness in flexion was not significantly different (P = 0.16). Number of cycles to failure was found significantly larger for the "Frame" technique (P = 0.01).

**Conclusions:**

In an in-vitro context the proposed method offers enhanced biomechanical stability and at the same time significantly reduces implant costs.

## Background

Fractures involving the distal humerus continue to challenge orthopedic surgery. Distal humeral fractures comprise approximately 2% of all fractures and one-third of all humeral fractures [1-3]. The risk of functional impairment following non-operative treatment is high [4-6]. On the other hand, establishing stability by internal fixation may be technically demanding in the face of complex fracture patterns and rising incidence of osteoporosis [2,7,8]. However, with respect to anatomic reduction, reconstitution of joint congruity, restoration of the functional bone axis, fixation stability and remobilization, it is generally accepted that internal fixation provides the most favorable outcome for distal humeral fractures [2,4-6,9,10]. Particularly in reduced bone quality, promising biomechanical and biological performance have been demonstrated when using angular stable Locking Compression Plates (LCP) with anatomical shape [11]. Disadvantages of LCP osteosynthesis are high costs and unavailability on some markets, especially in second and third world countries [2,8]. The LCP double plate osteosynthesis in 90° configuration has become one of the most popular treatment options mainly because of a less demanding surgical approach [9,12]. Besides plating in 90° fashion, alternative concepts involve parallel plate configurations, either locked or non-locked [13,14]. According to the theory of McKee and Jupiter [15], who figuratively compared distal humerus fixation to clamping a spool between thumb and index finger, the biomechanical benefit of interconnecting the two humeral columns is frequently emphasized in the literature [13,16]. Self et al. [17] proposed the use of an interconnection bolt linking two parallel plates together. O'Driscoll et al. [18] aimed to increase the stability of the repair construct by interdigitation of screws. In this study another method is proposed to join the humeral columns with cost-efficient conventional implants. The technique involves a conventional reconstruction plate in combination with a 1/3 tubular plate in a parallel mediolateral configuration. A transverse connecting screw is inserted from lateral to medial through the most distal hole of the tubular plate. Medially, the screw interlocks with the reconstruction plate to establish a solid connection. The proposed osteosynthesis in a "U-Frame" configuration decreases the implant costs markedly compared to an LCP based solution. Drawbacks of conventional fixation hardware, such as damage to the periosteal blood supply or the necessity for accurate plate contouring to avoid shortcomings in reduction, need to be weighed against the benefits. This study investigates the biomechanical performance of the proposed "Frame"-technique in a human cadaveric model and compares it to the 90° LCP osteosynthesis considered as one of today's predominant fixation techniques [12]. Our hypothesis was that the Frame construct would perform at least as well as the established LCP osteosynthesis under static and cyclic loading.

## Methods

Eight pairs of human cadaveric humeri (4 male, 4 female donors; mean age 88 years; range 79 - 96 years) were obtained from the department of Pathology, Kantonsspital Basel, Switzerland, where they had been harvested post mortem with appropriate consent of the relatives. Provision of the specimens was approved by the ethical commission of Kantonsspital Basel. The specimens were stored fresh frozen at -20°C. Bone mineral density (BMD) was measured for all samples in the cancellous bone of the distal condyles by means of peripheral quantitative computed-tomography using an Xtreme-CT (SCANCO Medical AG, Bassersdorf, Switzerland, resolution 82 μm). Left and right bones of each pair were randomly assigned to two study groups: 1) Frame-group; 2) LCP-group. Equal numbers of left and right specimens were assured in each group.

### Specimen preparation

All bones were thawed at room temperature and stripped of soft tissue. Before performing the operations, an intra-articular distal humerus fracture with metaphyseal comminution (AO type 13-C2.3) [19] was simulated. A 5 mm transverse gap was created 28 mm proximal to the most distal aspect of the joint surface. Additionally, an intra-articular fracture line was sawed proximally from the Trochlea notch splitting the condylar block. The osteotomy was then reduced by temporarily affixing the distal fragments with a reduction forceps and a transverse Kirschner (K-) wire. For anatomical reconstruction of the bone the transverse gap was temporarily filled with a 5 mm spacer while securing distal and proximal parts with additional K-wires. The spacer was removed after completing the osteosyntheses. All implants used in the study were manufactured by Synthes GmbH (Bettlach, Switzerland). Implant material was Titanium.

### Frame constructs

**Figure 1:**
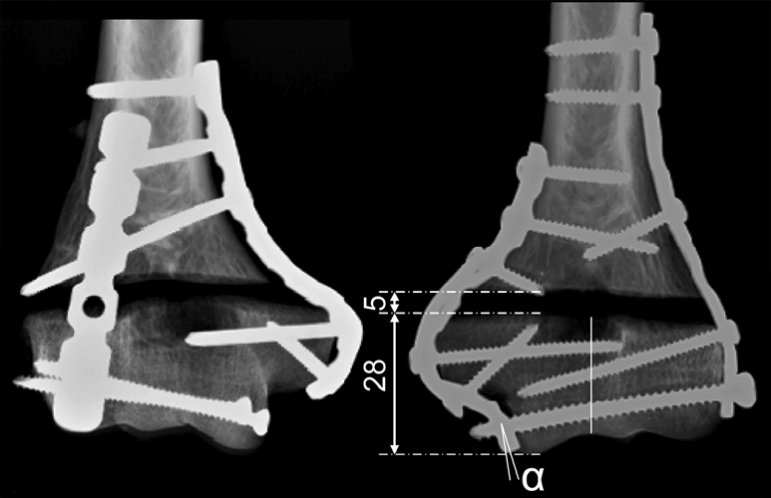
**Anteroposterior radiographs of an operated bone pair**. Left: LCP construct with 90° plate arrangement. Right: "Frame" technique with interconnection between both plates parallel to the joint surface for establishment of a "U"-shape configuration. The interconnection screw engages with the reconstruction plate. α indicates an oblique orientation of the plate, which further promotes the anchorage of the screw. Numbers are given in mm.

All Frame constructs were operated by a single experienced surgeon (E. R.). A 7-hole 3.5 mm 1/3 tubular plate was contoured to the lateral aspect of the reduced and temporarily stabilized bone. A conventional 7-hole, 3.5 mm reconstruction plate was contoured to the medial surface. Both plates were temporarily clamped to the bone. A 2.5 mm drill bit was placed into the most distal hole of the reconstruction plate. A drill channel was created parallel to the joint surface from medial to lateral, aiming at the most distal hole of the tubular plate in a free-hand manner. The plates were removed and the channel was then over-drilled from medial to lateral using a 5 mm drill bit in order to prevent screw to bone anchorage. A 4.5 mm cortex screw (self-tapping) was then inserted opposite to the drilling direction (from lateral to medial) through the most distal hole of the tubular plate into the transverse drill channel. Medially, the screw engaged with the most distal hole of the reconstruction plate similarly to a screw-nut connection. The diameter of the plate hole was 4.0 mm while the outer diameter of the screw thread was 4.5 mm. These dimensions allow insertion of the screw with moderate torque for stable connection of both elements. A slightly oblique orientation of the reconstruction plate with respect to the screw axis might further contribute to a rigid coupling at the screw-plate interface (Fig. 1). Plastic deformation of thread and plate hole is likely to occur. Since the components used were not engineered for this purpose, the assembly was a special object of our investigation. Careful compression was generated in the condylar block when tightening the interconnection screw. The screw length was determined such that the screw protruded approximately 5 mm from the medial cortex. The plates were further fixed to the condylar block by two additional 3.5 mm screws (self-tapping) medially and one laterally to provide rotational stability to the construct (anti-rotation screws). Proximally, both plates were secured to the diaphysis by inserting three 3.5 mm cortex screws (self-tapping) on either side. The two most proximal screws penetrated both cortices. For detailed screw configuration see Fig. 1.

### LCP constructs

The LCP operations were performed according to the established double plate osteosynthesis in 90°-configuration on the medial and posterolateral aspects of the humerus. An experienced senior resident surgeon (V. B.) performed all LCP operations following the AO guidelines [12]. 3.5 mm locking plates (posterolateral: 6-hole LCP reconstruction plate, medial: 7-hole LCP reconstruction plate) were individually contoured to the shape of the bone according to Jupiter [20]. Distally, each plate was equipped with two monocortical 3.5 mm locking screws. Proximally, three screws were inserted to fix each plate. All screws were tightened with a 1.5 Nm torque limiter (Synthes GmbH, Bettlach, Switzerland). Anteroposterior radiographs of an instrumented bone-pair are shown in Fig. 1.

### Mechanical testing

**Figure 2:**
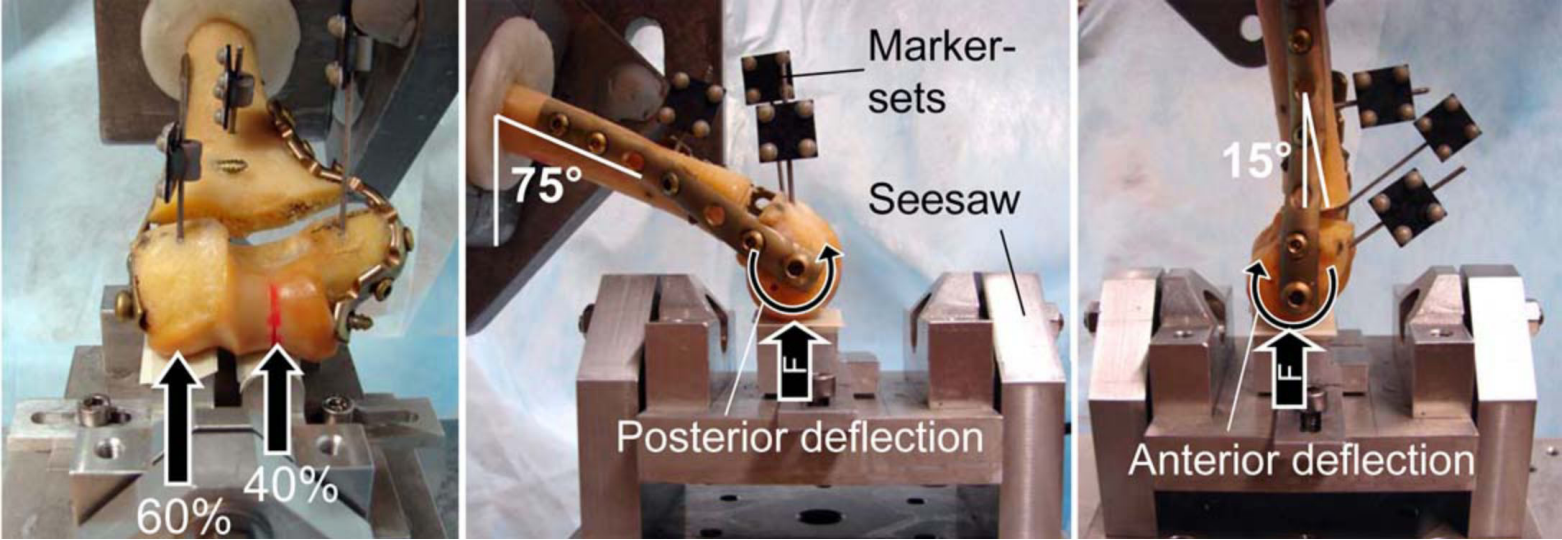
**Test setup**. The specimen is placed on a seesaw for physiological force transmission. Marker-sets for optical motion tracking are attached to all fragments. Left and middle: Setup for flexion test with angulation of the shaft of 75° to the vertical. Right: Setup for extension test with 15° angulation to the vertical. The vertical reaction force of the seesaw is indicated by F.

Generally, the methodology for biomechanical testing was based on an earlier publication with certain modifications [11]. The specimens were cut proximally to a total length of 160 mm. 60 mm of the proximal end were embedded in Polymethylmethacrylate (Beracryl, W. Troller AG, Fulenbach, Switzerland) to fix the specimen to the actuator of a servo-hydraulic testing machine (MTS 858 Minibionix II, MTS Systems, Minneapolis, MN, USA, 4 kN loadcell). Distally, the Capitellum and Trochlea notch rested on a seesaw with two anatomically shaped supports covered with a layer of silicone to avoid peak stress at the contact points. Eccentric positioning of the supports with respect to the rotational axis of the seesaw enabled physiological force distribution of 60% at the Capitellum and 40% at the Trochlea [11,21,22] (Fig. 2). A cross-table was positioned below the seesaw to eliminate shear forces.

**Figure 3:**
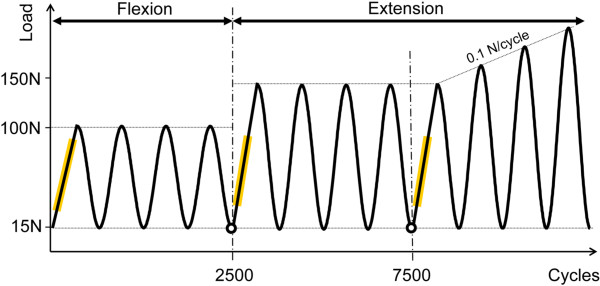
**Testing protocol**. First 2500 test-cycles were performed in flexion between 15 and 100 N. Cycles 2500 to 7500 were performed in extension between 15 and 150 N. To provoke fatigue failure, the load was then monotonically increased at 0.1 N/cycle. Stiffness was evaluated from quasi-static ramps at 0, 2500 and 7500 cycles as indicated by yellow bars.

All specimens were tested successively in simulated flexion and extension of the arm. For the flexion test the functional axis of the humerus was rotated 75° to the vertical towards posterior (Fig. 2). At the beginning of the test, a quasi-static loading ramp was applied at 15 N/s with a vertical force vector to assess construct stiffness. Sinusoidal loading was then performed between 15 N and 100 N for 2500 cycles. Subsequently, the humeral shaft angle was reduced to 15° simulating extension (Fig. 2). Another quasi-static loading ramp was applied and additional 5000 load cycles of 15 N to 150 N were performed. In case no severe failure of the construct occurred, construct stiffness was again measured with a quasi-static ramp and cyclic loading was continued with monotonically increasing peak force (0.1 N/cycle) [23] until severe failure of the sample became obvious. The load valley was maintained constant at 15 N. All cyclic tests were carried out at 2 Hz. The testing protocol is visualized in Fig. 3.

Throughout the experiment the deformation of the construct was monitored by means of an optical motion capturing system (Qualisys ProReflex MCU, Qualisys AB, Gothenburg, Sweden). Sets of retro-reflective markers were attached to the humeral shaft and to both distal fragments to track the fragment motions in six degrees of freedom (Fig. 2). For the Frame constructs, the connection between transverse screw and reconstruction plate was monitored by video observation throughout the experiment. To identify the mode of failure, radiographs were taken post-operation and after testing was completed.

### Data evaluation and statistics

Construct stiffness was determined from the quasi-static loading ramps as the slope of the load - displacement curves. The deflection of the distal fragments with respect to the shaft in the sagital plane was identified as the predominant deformation of the investigated constructs. Deflections were computed from the motion tracking data throughout the cyclic tests at minimum load to identify plastic construct deformation. From pilot experiments, a deflection angle of ± 3° of the lateral fragment appeared appropriate to quantify the point of failure for statistical evaluation. The number of cycles to 3° sagital deflection was determined for all specimens. Additionally, the magnitude of spatial displacement at the intra-articular gap was evaluated from the motion tracking data. To assess differences between study groups, non-parametric paired test statistics (Wilcoxon signed ranks) were employed on BMD measurements, on cycles to 3° deflection and on the stiffness measurements. Level of significance was α = 0.05.

## Results

Bone mineral density was 0.48 ± 0.16 g/cm^3 ^(mean ± SD) for the Frame-group and 0.49 ± 0.15 g/cm^3 ^for the LCP-group. This difference was not significant between study groups (P = 0.73).

**Figure 4:**
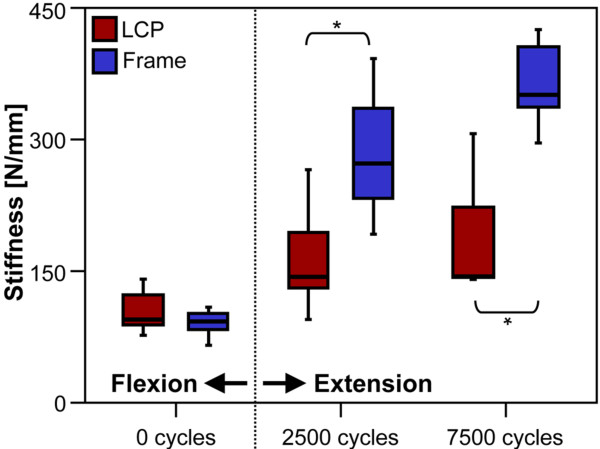
**Construct stiffness**. Stiffness obtained from quasi-static measurements for both study-groups at the beginning of the cyclic test in flexion, at the beginning of the cyclic extension test (after 2500 cycles) and after 7500 cycles. * indicates statistical significance.

Construct stiffness in flexion as obtained from the quasi-static ramps at the beginning of the test was 91 ± 5 N/mm for the Frame constructs and 103 ± 8 N/mm for the LCP samples. This difference was not significant between groups (P = 0.16). The stiffness in extension (at the beginning of the extension test) was significantly higher for the Frame-group (281 ± 25 N/mm) compared to the LCP-group (161 ± 21 N/mm) (P = 0.01; Fig. 4). After 7500 cycles (5000 cycles of these in extension) this difference was still significant (P = 0.02).

**Figure 5:**
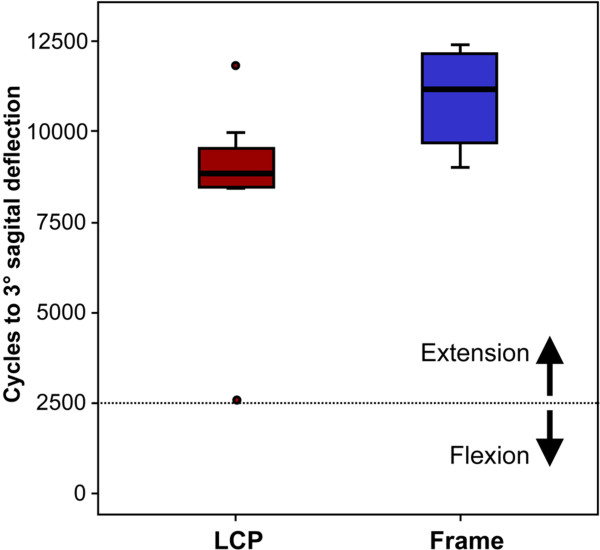
**Cycles to 3° deflection**. Number of cycles to 3° deflection in the sagital plane for both study groups.

All specimens survived the cyclic flexion test. Within the following 5000 test-cycles in extension one LCP specimen (lowest BMD of the test) exceeded the limit of 3° sagital deflection. Mean number of cycles to 3° deflection was 11001 ± 473 for the Frame samples and 8505 ± 935 for the LCP specimens. This difference was significant between groups (P = 0.01; Fig. 5). The corresponding load levels at 3° deflection were 529 ± 49 N (Frame) and 248 ± 31 N (LCP). The magnitude of the gap displacement after 7500 cycles was at a maximum 0.09 mm for the Frame-group and 0.18 mm for the LCP-group (measured at minimum load).

Failure modes were identified based on radiographs and visual inspection after testing was completed (Tab. 1). Six LCP specimens demonstrated pull-out of the screws placed in the lateral fragment. The two specimens with the highest BMD failed due to bending of the posterolateral plate, with no sign of screw loosening. All Frame constructs failed due to bending of the transverse interconnection screw together with bending of the tubular plate. No twisting of the plates was found. In three cases additional loosing of the lateral anti-rotation screw was observed. No failures occurred at the proximal fragment. No obvious failures occurred at the connection between transverse interconnection screw and reconstruction plate. The screw could easily be removed with a screwdriver.

**Table 1:** Failure modes

Pair	BMD [g/cm^3^]		Failure mode	
		
	Frame	LCP	Frame	LCP
1	0.30	0.28	Bending connection screw and tubular plate	Screw pull-out
2	0.28	0.33	Bending connection screw, loosening anti-rotation screw	Screw pull-out
3	0.42	0.36	Bending connection screw and tubular plate	Screw pull-out
4	0.47	0.48	Bending connection screw and tubular plate	Screw pull-out
5	0.49	0.56	Bending connection screw, loosening anti-rotation screw	Screw pull-out
6	0.55	0.57	Bending connection screw, loosening anti-rotation screw	Screw pull-out
7	0.54	0.59	Bending connection screw, loosening anti-rotation screw	LCP bending
8	0.81	0.72	Bending connection screw and tubular plate	LCP bending

Bone Mineral Densities (BMD) and failure modes for all specimens.

## Discussion

The clinical outcome of distal humeral fractures is variable due to complex fracture pattern, osteoporosis, high loading exposure and long moment arms. Several osteosynthetic strategies are available and in frequent use. Angularly stable concepts have shown particularly promising results in osteoporotic bone [11]. However, costs are high due to demanding production processes and advanced standards. The underlying idea of the alternative proposed in this study relates to the principle of interconnecting the humeral columns to enhanced stability [13,16,18]. From biomechanical findings Korner et al. [8] concluded that the implant configuration might be more important than the implant type in distal humeral osteosynthesis. In the aircraft industry, for example, the same principle applies to light weight constructions, where the mechanical properties of the individual element are less important than the geometric arrangement of all components. In agreement with the theory of McKee and Jupiter [15], who considered securing the Trochlea between two bony columns to be most important for stable fixation, the proposed technique unites these columns to establish a triangular configuration for superior load bearing. A comparable approach was published by Self et al. [17] using connection bolts and nuts. They reported promising stability but at the same time stated soft tissue problems related to prominent fixation hardware.

This in-vitro study compared the biomechanical stability of the described "Frame"-technique, in a low-profile fashion for reduced soft-tissue irritation, with the established LCP osteosynthesis in 90° configuration. To obtain an overall impression of the function of these constructs under different loading conditions, a cyclic test in simulated flexion of the arm was followed by cyclic loading in extension until failure occurred. Clinically, distal screw pull-out at the lateral column is frequently reported for angular stable plating in 90° configuration [8,18]. This is likely to happen in extension because of predominant anterior bending in the sagital plane (Fig. 2). According to mechanical principles, the condylar block is deflected towards anterior in extension while in flexion the fragments bend posteriorly. We used isolated humeri for testing [8,11,24-27]. The distal articulation was modelled by two anatomical supports simulating physiological force transmission [11,21]. Accentuated loading on the lateral side generated a natural valgus bending moment. In the healthy elbow, physiological forces between 0.3 and 0.5 times bodyweight (approx. 210 - 350 N) are reported during routine activities [28]. Previous biomechanical studies used loading regimes between 100 and 250 N [11,18,29] to test repair constructs. For the initial cyclic flexion test we chose a comparatively low load level of 100 N to avoid severe failure. For the subsequent extension test the load was increased to 150 N to enable comparison with the study of Schuster et al. [11]. Since no failure could be provoked during the first 7500 cycles the loading amplitude was then continuously increased to failure according to the protocol of Windolf et al. [23]. Loading of the humerus is complex. Torsion and other loading modes were not considered here and would require further testing to draw an overall conclusion.

Compared to the LCP technique, superior fatigue properties were demonstrated for the Frame construct in reduced bone quality. According to Schuster et al. [11], who proposed a BMD threshold < 0.46 g/cm^3 ^to define poor bone stock in the distal humerus, 3 out of 8 pairs fell below this limit. The mean BMD (0.48 g/cm^3^) was slightly higher but still comparable to the study of Schuster et al. Construct stiffness was comparable to values reported in the literature [11,18,27,29]. Both study groups revealed similar stiffness in flexion. In extension, construct stiffness was found significantly higher for the Frame technique. A potential effect of reduced motion in the fracture gap on bone healing [30], either accelerating or prolonging, can not be evaluated here. A maximum observed deformation of the intra-articular gap of 0.2 mm for all specimens, indicates sufficient stabilization of the condylar block regardless of the fixation method.

Screw pull-out in the lateral column was observed for the majority of LCP specimens, which is recognized as a predominant failure pattern clinically. The Frame samples showed partial loosening of the lateral anti-rotation screw (Fig. 1). This might be an indicator for a potential weakness of the technique, since the interconnection screw does not contribute to the rotational stability of the condylar block. The observed bending of the tubular plate on the lateral side might be the result of higher forces transferred via the Capitellum compared to the Trochlea [11,21,22]. A tubular plate was chosen because, 1) it offers a low profile under a rather thin soft tissue layer, and 2) it provides a clearance fit for a 4.5 mm screw. However, other options such as the use of another reconstruction plate could be considered. All Frame specimens showed consistent bending of the transverse interconnection screw. The absence of screw-to-bone engagement inside the channel might allow alternating motion of the screw, causing enlargement of the through-hole by wear. The connection between the transverse screw and the reconstruction plate was noted as potential weak point. However, no failures occurred here. There is also the potential for sharp metal chips to form due to motion between the screw and the thread-less through-hole. No such debris was observed here.

Stability of the condylar block fixation relies on compression generated between the medial and lateral plates. Disturbing effects of plate-bone compression on blood circulation and hence on the healing process have been reported [31]. Angularly stable plating with reduced contact might have biological advantages at the distal humerus in terms of accelerated fracture healing. Further investigation in an in-vivo context is needed. In clinical practice, application of the Frame technique might be technically more demanding than other fixation methods of the distal humerus. In particular, drilling the through-hole parallel to the joint surface in a free-hand manner carries a risk of articular penetration and hence, requires careful technique. However, in our in-vitro environment no complications occurred. Clinically, transposition of the ulnar nerve is recommended since nerval interference by the transverse fixation screw may be possible. When the osteosynthesis is removed, the nerve is again at risk and requires special attention.

## Conclusions

In this study a method for treatment of distal humeral fractures is proposed using conventional implants in a new configuration. The technique offers the advantage of significantly reduced implant costs compared to angularly stable plating. Both, stiffness and stability under cyclic loading were found to be superior to the LCP osteosynthesis with 90° plate arrangement. Despite the need for further evaluation in a clinical environment, the concept offers a promising alternative for distal humeral fracture treatment.

## Competing interests

The authors declare that they have no competing interests.

## Authors' contributions

MW planned the study developed the test setup, performed the measurements, carried out the statistics and drafted the manuscript. ERM developed the idea of the study and performed the operations. VB performed the operations and helped with drafting the manuscript. BG participated in the design of the study and evaluated the data. KS participated in the study design and helped to draft the manuscript. All authors read and approved the final manuscript.

## Pre-publication history

The pre-publication history for this paper can be accessed here:

http://www.biomedcentral.com/1471-2474/11/172/prepub
